# *CXCL9*-*11* polymorphisms are associated with liver fibrosis in patients with chronic hepatitis C: a cross-sectional study

**DOI:** 10.1186/s40169-017-0156-3

**Published:** 2017-07-28

**Authors:** María Ángeles Jiménez-Sousa, Ana Zaida Gómez-Moreno, Daniel Pineda-Tenor, Luz Maria Medrano, Juan José Sánchez-Ruano, Amanda Fernández-Rodríguez, Tomas Artaza-Varasa, José Saura-Montalban, Sonia Vázquez-Morón, Pablo Ryan, Salvador Resino

**Affiliations:** 1 Unidad de Infección Viral e Inmunidad, Centro Nacional de Microbiología, Instituto de Salud Carlos III, Carretera Majadahonda- Pozuelo, Km 2.2, 28220 Majadahonda, Madrid, Spain; 2 Servicio de Aparato Digestivo, Hospital Virgen de la Salud, Toledo, Spain; 3 Servicio de Laboratorio Clínico, Hospital Universitario de Fuenlabrada, Madrid, Spain; 4 Servicio de Laboratorio Clínico, Hospital Virgen de la Salud, Toledo, Spain; 5 Servicio de Medicina Interna, Hospital Universitario Infanta Leonor, Madrid, Spain

**Keywords:** Liver stiffness, Chronic hepatitis C, Cirrhosis, CXCL9-11, SNPs, Liver fibrosis

## Abstract

**Background and aims:**

*CXCL9*-*11* polymorphisms are related to various infectious diseases, including hepatitis C virus (HCV) infection. In this study, we analyzed the association between *CXCL9*-*11* polymorphisms and liver fibrosis in HCV-infected patients.

**Methods:**

We performed a cross-sectional study in 389 patients who were genotyped for *CXCL9*-*11* polymorphisms (*CXCL9* rs10336, *CXCL10* rs3921, and *CXCL11* rs4619915) using the Sequenom’s MassARRAY platform. The primary outcome variable was the liver stiffness measurement (LSM). We established three cut-offs of LSM: LSM ≥ 7.1 kPa (F ≥ 2—significant fibrosis), LSM ≥ 9.5 kPa (F ≥ 3—advanced fibrosis), and LSM ≥ 12.5 kPa (F4—cirrhosis).

**Results:**

Recessive, overdominant and codominant models of inheritance showed significant values, but the overdominant model was the best fitting our data. In this case, *CXCL9* rs10336 AG, *CXCL10* rs3921 CG and *CXCL11* rs4619915 AG were mainly associated with lower values of LSM [(adjusted GMR (aGMR) = 0.85 (p = 0.005), aGMR = 0.84 (p = 0.003), and aGMR = 0.84 (p = 0.003), respectively]. Patients with *CXCL9* rs10336 AG genotype had lower odds of significant fibrosis (LSM ≥ 7.1 kPa) [adjusted OR (aOR) = 0.59 (p = 0.016)], advanced fibrosis (LSM ≥ 9.5 kPa) [aOR = 0.54 (p = 0.010)], and cirrhosis (LSM ≥ 12.5 kPa) [aOR = 0.56 (p = 0.043)]. Patients with *CXCL10* rs3921 CG or *CXCL11* rs4619915 AG genotypes had lower odds of significant fibrosis (LSM ≥ 7.1 kPa) [adjusted OR (aOR) = 0.56 (p = 0.008)], advanced fibrosis (LSM ≥ 9.5 kPa) [aOR = 0.55 (p = 0.013)], and cirrhosis (LSM ≥ 12.5 kPa) [aOR = 0.57 (p = 0.051)]. Additionally, *CXCL9*-*11* polymorphisms were related to lower liver stiffness under a codominant model of inheritance, being the heterozygous genotypes also protective against hepatic fibrosis. In the recessive inheritance model, the *CXCL9* rs10336 AA, *CXCL10* rs3921 CC and *CXCL11* rs4619915 AA were associated with higher LSM values [(adjusted GMR (aGMR) = 1.19 (p = 0.030), aGMR = 1.21 (p = 0.023), and aGMR = 1.21 (p = 0.023), respectively]. Moreover, patients with *CXCL9* rs10336 AA genotype had higher odds of significant fibrosis (LSM ≥ 7.1 kPa) [adjusted OR (aOR) = 1.83 (p = 0.044)] and advanced fibrosis (LSM ≥ 9.5 kPa) [aOR = 1.85 (p = 0.045)]. Furthermore, patients with *CXCL10* rs3921 CC or *CXCL11* rs4619915 AA genotypes had higher odds of advanced fibrosis (LSM ≥ 9.5 kPa) [aOR = 1.89 (p = 0.038)].

**Conclusions:**

CXCL9-*11* polymorphisms were related to likelihood of having liver fibrosis in HCV-infected patients. Our data suggest that *CXCL9*-*11* polymorphisms may play a significant role against the progression of CHC and could help prioritize antiviral therapy.

## Background

Hepatitis C virus (HCV) leads to the development of chronic liver disease or liver-related death worldwide [1, 2]. Around 80% of the subjects exposed to the HCV usually develop chronic hepatitis C (CHC), which may result in significant liver fibrosis, cirrhosis and end stage liver disease [3]. Moreover, CHC constitutes a major indication for liver transplantation in the United States [4] and Europe [5]. However, the impact of HCV infection is highly variable, ranging from minimal rates of fibrosis progression to extensive fibrosis and cirrhosis in a few years [6]. Additionally, HCV antiviral therapy may reduce the clinical consequences of CHC, but patients with cirrhosis, despite HCV eradication, remain at risk of disease progression [7–9].

The management of patients with CHC depends on their clinical stage [10], since patients without cirrhosis have a much longer median survival time than those with cirrhosis [6]. Thus, early recognition of patients with CHC at high risk for developing liver fibrosis and cirrhosis is important, because it ensures optimal preventive management strategies that can modify the course of CHC disease [11]. The hepatic biopsy is the gold standard test to diagnose and quantify liver fibrosis, but this procedure is associated with several drawbacks [12, 13]. Liver stiffness measurement (LSM) using transient elastography is a noninvasive method based on liver elasticity observations, which may accurately predict the presence of fibrosis/cirrhosis in patients with CHC [12, 13].

The pathogenic mechanisms of the accelerated progression of liver injury are incompletely understood; but single nucleotide polymorphisms (SNPs), liver inflammatory profile and the impaired immune response are thought to be important determinants of the evolution of liver disease [14]. In this context, the lymphocyte migration from the periphery to liver parenchyma mediated by chemokines constitutes a major event for development of liver fibrosis in response to HCV infection [15]. CXCL chemokine ligand (CXCL) 9 [monokine induced by interferon γ (Mig)], CXCL10 [interferon-γ—inducible protein-10 (IP-10)], and CXCL11 (interferon-inducible T cell α chemoattractant; I-TAC) are produced in the liver by infected hepatocytes during CHC. This response induces migration of activated T cells (T-helper/T-cytotoxic type-1 cell (Th1/Tc1) response) from the periphery to the infected liver parenchyma via chemokine (C-X-C motif) receptor 3 [16]. It has been reported an increase of CXCL9 expression in hepatocytes of HCV-infected patients, and a correlation between CXCL9 levels and liver fibrosis [17, 18]. However, an anti-fibrotic role of CXCL9 has also been described [19–21]. Besides, increased CXCL10 levels have been associated with liver injury in HCV-infected patients [17, 21–26], and CXCL11 expression levels have been related to both portal and lobular inflammation; a clear relationship with liver disease in CHC has not been found [17, 22, 27]. Additionally, CXCL9-11 chemokines have been significantly correlated with the level of hepatoprotective cytokines such as IL6 and IL10, suggesting their involvement in a counter-regulatory response during the progression of liver disease [17].

*CXCL9*-*11* polymorphisms have been related to severity in various infectious diseases such as those caused by enterovirus-71 [28], hepatitis B infection [29, 30], malaria [31], Chagas disease [32], and tuberculosis [33]. Besides, we have found an association between *CXCL9*-*11* polymorphisms and liver disease in human immunodeficiency virus (HIV)/HCV-coinfected patients [34]. These patients have a damaged immune system due to HIV infection, causing hepatitis C virus to evolve more aggressively [35, 36]. However, to our knowledge, there is no information regarding the relationship between *CXCL9*-*11* polymorphisms and liver disease in HCV-monoinfected patients.

In the present study, we analysed the association between *CXCL9*-*11* polymorphisms and liver fibrosis, considering LSM values, in HCV-infected patients.

## Patients and methods

### Patients

We carried out a cross-sectional study on 389 HCV-infected patients who had liver stiffness assessed by transient elastometry in Virgen de la Salud Hospital (Toledo, Spain) between 2005 and 2015. The study was conducted in accordance with the Declaration of Helsinki, all patients gave their written consent for the study, and the Research Ethic Committee (“Comité de Ética de la Investigación”) approved the study.

The selection criteria were: (1) detectable HCV RNA by polymerase chain reaction; (2) availability of a valid LSM; (3) availability of DNA sample. The exclusion criteria were: (1) co-infection with hepatitis B virus or human immunodeficiency virus; (2) clinical evidence of hepatic decompensation at enrollment or a prior history of hepatic decompensation; and (3) hepatocellular carcinoma at enrollment or a previous history of hepatocellular carcinoma.

### Clinical and laboratory data

Clinical and epidemiological data were obtained from medical records. The time since HCV diagnosis was calculated starting from the diagnosis date to the date of last visit in our study. High alcohol intake was considered to be ≥20 grams/day in women and ≥60 grams/day in men [37]. Patients could have been treated with HCV therapy [peg-Interferon (IFN)-α/ribavirin] according to clinical guidelines [38, 39]. However, we only included non-responder patients with detectable serum HCV-RNA.

### Liver stiffness measurement

Liver stiffness measurement was assessed by transient elastography (FibroScan^®^, Echosens, Paris, France). Results were expressed in kilopascals (kPa) with a range of 2.5–75 kPa. Transient elastography was performed by a trained operator (medical doctor), and measurements were considered reliable when the interquartile-range-to-median ratio was <0.30 [40]. For at least ten successful measurements.

### Outcome variable

The primary outcome variable was the LSM examined as continuous and categorical variable. Three different cut-offs of LSM [41], were established to stratify all patients: LSM ≥ 7.1 kPa (F ≥ 2—significant fibrosis), LSM ≥ 9.5 kPa (F ≥ 3—advanced fibrosis), and LSM ≥ 12.5 kPa (F4—cirrhosis).

### Genotyping of CXCL9-11 polymorphisms

We analyzed three SNPs within the *CXCL9*-*11* family (*CXCL9* rs10336, *CXCL10* rs3921 and *CXCL11* rs4619915) [34, 42]. DNA samples were genotyped at the Spanish National Genotyping Center (CeGen-PRB2; http://www.usc.es/cegen/) by using the iPLEX^®^ Gold technology and Agena Bioscience’s MassARRAY platform (San Diego, CA, USA).The quality control was performed according to the CeGen criteria, which includes a genotyping call-rate over 95% for all the SNPs; duplicated samples on each plate to check for technical replicates; a negative control; and three positive Coriell controls from Human Genetic Cell Repository (NA10860, NA10861 and NA11984). These controls were included on each batch to exclude DNA contamination and ensure a technically correct laboratory process, respectively.

A control healthy population was obtained the at the 1000 Genomes Project’s website (http://www.1000genomes.org/home): This database provides a broad representation of common human genetic variation detected by next generation sequencing in multiple populations [43]. Our control population was the Iberian population in Spain (IBS), which includes 107 individuals.

### Statistical analysis

To analyze the differences among groups according to *CXCL9*-*11* genotypes, Mann–Whitney U test and Kruskal–Wallis were used for continuous variables and Chi square test or Fisher’s exact test for categorical variables.

For the genetic association study, generalized linear model (GLM) was used to analyze the outcome variables with *CXCL9*-*11* polymorphisms. This analysis was carried out according to dominant, codominant, overdominant, recessive and additive models. These analyses were tested according to the goodness of fit evaluated by Akaike information criterion (AIC) value and Bayesian information criterion (BIC). On the one hand, GLM with a gamma distribution was used to investigate the association between *CXCL9*-*11* polymorphisms and the LSM (continuous variable). Besides, natural logarithmic transformation (Ln) of LSM was used due to a skewed distribution. The GLM gives the differences between groups and the geometric mean ratio (GMR), or the ratio by which the geometric mean of the original outcome is multiplied. On the other hand, GLM with binomial distribution (logit-link) was used to investigate the association of *CXCL9*-*11* polymorphisms with categorical outcome variables (F ≥ 2, F ≥ 3, and F4). This test gives the differences between groups and the odds ratio (OR) for categorical outcome variables. For univariate model, we included each SNP separately. For multivariate model, each SNP together with all available relevant covariates (multivariate model) were included using the Enter algorithm (Forced Entry). The covariates that we have had available for inclusion in our study were age, gender, time since HCV diagnosis, HCV genotype, injection drug use, high alcohol intake, and non-responder patients to HCV antiviral therapy prior to baseline (patients who failed therapy). All statistical tests were performed with the Statistical Package for the Social Sciences (SPSS) 21.0 software (IBM Corp., Chicago, USA). All p values were two-tailed and statistical significance was defined as p value <0.05.

In addition, Hardy–Weinberg equilibrium (HWE) and pairwise linkage disequilibrium (LD) were analyzed by Haploview 4.2 software. Haplotype-based association test was performed with Plink software (http://pngu.mgh.harvard.edu/~purcell/plink/).

## Results

### Patient characteristics

Table 1 shows the characteristics of 389 chronic HCV-infected patients, 71 were cirrhotic (LSM ≥ 12.5 kPa). In brief, 58.6% were male, the median age was 49.6 years, 19.3% reported a high intake of alcohol, 17% were prior injection drug users, 81.2% were infected by HCV genotype 1, and 20.8% were patients treated with antiviral therapy (peg-IFN-α/ribavirin) who did not achieved sustained virological response (non-responders). The median LSM was 6.9 kPa and the median time since HCV diagnosis was 8.6 years.

**Table 1:** Clinical and epidemiological characteristics of HCV-infected patients

Characteristics	Data
No.	389
Male sex	228 (58.6%)
Age (years)	49.6 (43.2; 57.7)
Time of HCV diagnosis (years)	8.6 (2.1; 14.1)
High alcohol intake	75 (19.3%)
Prior injection drug use	66 (17%)
HCV genotype (n = 383)	
1	311 (81.2%)
2	5 (1.3%)
3	42 (11%)
4	23 (6%)
5	2 (0.5%)
Non-responder (peg-IFN-α/RBV)	81 (20.8%)
Liver stiffness (kPa)	6.9 (5.5; 10.5)
F0–F1 (<7.1)	205 (52.7%)
F2 (7.1–9.4)	65 (16.7%)
F3 (9.5–12.4)	48 (12.3%)
F4.1 (12.5–21.4)	41 (10.5%)
F4.2 (≥21.5)	30 (7.7%)

Values expressed as absolute numbers (%) and median (percentile 25; percentile 75)

*HCV* hepatitis C virus, *LSM* liver stiffness measure, *kPa* kilopascal, *IFN* interferon, *peg*-IFN-α/RBV pegilated-interferon-alpha/ribavirin

### CXCL9-11 polymorphisms

Table 2 shows allelic and genotypic frequencies of *CXCL9*-*11* polymorphisms. All SNPs had a minimum allele frequency >5%, displayed less than 5% of missing values, and were in HWE (p > 0.05). No significant differences were found between studied patients and IBS population. In addition, the allelic and genotypic frequencies in our dataset were in accordance with data listed on the NCBI SNP database for European population (http://www.ncbi.nlm.nih.gov/projects/SNP/).

**Table 2:** Summary of Hardy–Weinberg Equilibrium test and allelic and genotypic frequencies genotypes for *CXCL9*-*11* polymorphisms in HCV-infected patients compared to Iberian population (data from HapMap) (http://browser.1000genomes.org/index.html)

SNPs	HCV cohort (n = 389)					IBS group (n = 107)					χ^2^ test^a^	χ^2^ test^b^
	HWE	Alleles		Genotype		HWE	Alleles		Genotype		p value	p value
*CXCL9* rs10336	0.738	A	41%	AA	16%	0.677	A	45%	AA	22%	0.568	0.556
		G	59%	AG	50%		G	55%	AG	46%		
				GG	34%				GG	32%		
*CXCL10* rs3921	0.841	C	41%	CC	16%	0.884	C	45%	CC	21%	0.568	0.653
		G	59%	CG	49%		G	55%	CG	47%		
				GG	35%				GG	32%		
*CXCL11* rs4619915	0.841	A	41%	AA	16%	0.884	A	44%	AA	21%	0.668	0.650
		G	59%	AG	49%		G	56%	AG	45%		
				GG	35%				GG	34%		

Statistical: p values were calculated by Chi squared test

*HWE* Hardy–Weinberg Equilibrium, *HCV* hepatitis C virus, *IBS* Iberian populations in Spain, *CXCL* chemokine (C-X-C motif) ligand

^a^Differences between allele frequencies

^b^Differences between genotype frequencies

**Fig. 1:**
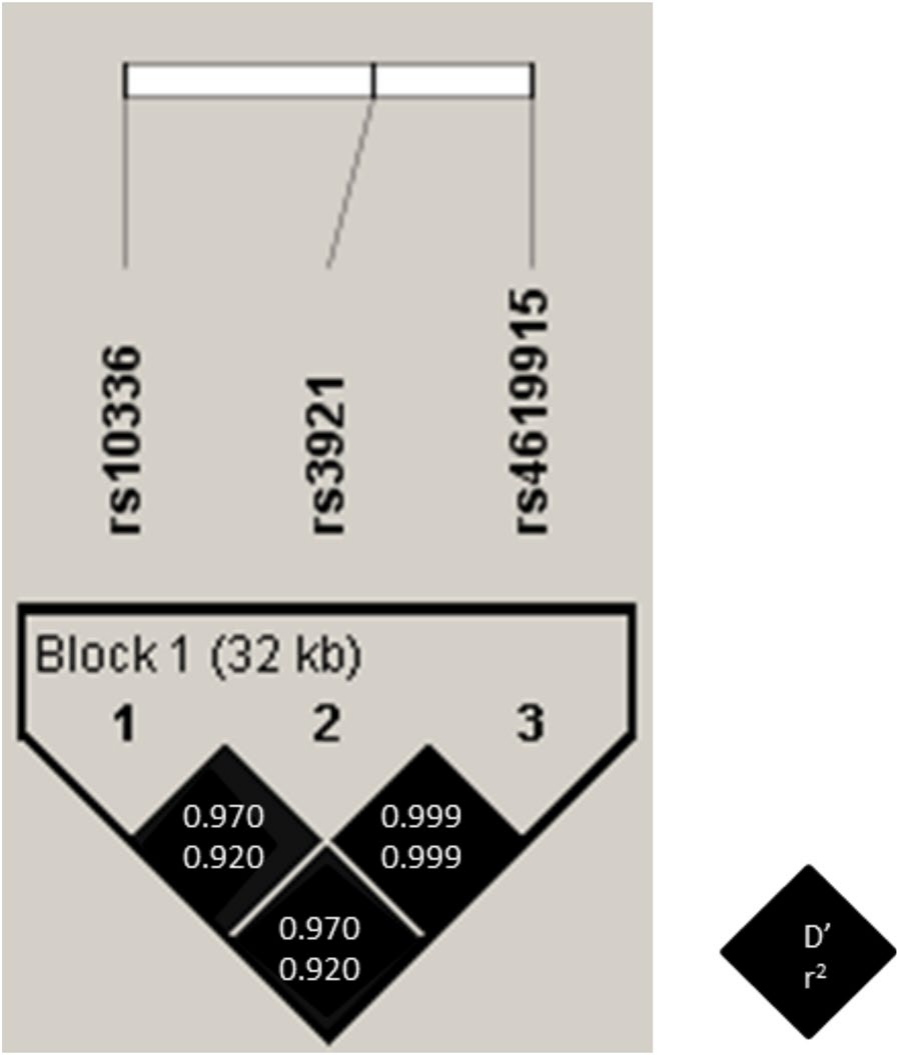
Pairwise linkage disequilibrium (LD) pattern for *CXCL9*-*11* polymorphisms. Each diagonal represents a different SNP, with each square representing a pairwise comparison between two SNPs

A strong LD among *CXCL9*-*11* SNPs was found (Fig. 1; D’ ≥ 0.97), meaning that there is no evidence of recombination between these SNPs. Furthermore, the R-squared among *CXCL9*-*11* SNPs was also high (Fig. 1; R-squared ≥ 0.92), indicating that these polymorphisms could substitute each other.

### CXCL9-11 polymorphisms and liver stiffness

The values of LSM and fibrosis stages according to *CXCL9*-*11* polymorphisms are shown in Table 3. The *CXCL9* rs10336 AG, *CXCL10* rs3921 CG and *CXCL11* rs4619915 AG were linked to lower values of LSM (p ≤ 0.002) and frequencies of F ≥ 2 (LSM ≥ 7.1 kPa; p ≤ 0.004), F ≥ 3 (LSM ≥ 9.5 kPa; p ≤ 0.003) and F4 (LSM ≥ 12.5 kPa; p ≤ 0.038) than patients with homozygote genotypes.

**Table 3:** Summary of LSM and fibrosis stages according to *CXCL9*-*11* polymorphisms (*CXCL9* rs10336, *CXCL10* rs3921, and *CXCL11* rs4619915) in HCV-infected patients

rs10336	AA	AG	GG	p^a^
No.	64	193	132	
LSM (kPa)	8.6 (6.1; 12.9)	6.7 (5.3; 8.9)	7.5 (5.6; 11.7)	*0.002*
F ≥ 2 (≥7.1)	40 (62.5%)	77 (39.9%)	67 (50.8%)	*0.004*
F ≥ 3 (≥9.5)	28 (43.8%)	44 (22.8%)	47 (35.6%)	*0.002*
F4 (≥12.5)	17 (26.6%)	26 (13.5%)	28 (21.2%)	*0.035*

rs3921	CC	CG	GG	p^a^
No.	63	191	135	
LSM (kPa)	8.6 (6.1; 12.9)	6.7 (5.3; 9.1)	7.6 (5.6; 11.6)	*0.001*
F ≥ 2 (≥7.1 kPa)	39 (61.9%)	75 (39.3%)	70 (51.9%)	*0.003*
F ≥ 3 (≥9.5 kPa)	28 (44.4%)	44 (23%)	47 (34.8%)	*0.003*
F4 (≥12.5 kPa)	17 (27%)	26 (13.6%)	28 (20.7%)	*0.038*

rs4619915	AA	AG	GG	p^a^
No.	63	191	135	
LSM (kPa)	8.6 (6.1; 12.9)	6.7 (5.3; 9.1)	7.6 (5.6; 11.6)	*0.001*
F ≥ 2 (≥ 7.1)	39 (61.9%)	75 (39.3%)	70 (51.9%)	*0.003*
F ≥ 3 (≥ 9.5)	28 (44.4%)	44 (23.0%)	47 (34.8%)	*0.003*
F4 (≥ 12.5)	17 (27%)	26 (13.6%)	28 (20.7%)	*0.038*

*p value* level of significance *LSM* liver stiffness measure, *kPa* kilopascal, *F* *≥* *2* significant fibrosis, *F* *≥* *3* advance fibrosis, *F4* cirrhosis, *CXCL* chemokine (C-X-C motif) ligand

Statistical: ^a^ p values were calculated by KW (continuous variable) or Chi squared test (dichotomous variable). Statistically significant differences are shown in italic

The association analysis was tested under five different genetic models of inheritance. Recessive, overdominant and codominant models showed significant values, but the overdominant model was the best fitting our data according to AIC and BIC.

The overdominant inheritance model was the model that best fitted to our data, and the association values are shown in Table 4. In this case, we also found that *CXCL9* rs10336 AG, *CXCL10* rs3921 CG and *CXCL11* rs4619915 AG were mainly associated with lower values of LSM [(adjusted GMR (aGMR) = 0.85 (p = 0.005), aGMR = 0.84 (p = 0.003), and aGMR = 0.84 (p = 0.003), respectively]. Moreover, patients with *CXCL9* rs10336 AG genotype had lower odds of significant fibrosis (LSM ≥ 7.1 kPa) [adjusted OR (aOR) = 0.59 (p = 0.016)], advanced fibrosis (LSM ≥ 9.5 kPa) [aOR = 0.54 (p = 0.010)], and cirrhosis (LSM ≥ 12.5 kPa) [aOR = 0.56 (p = 0.043)]. Furthermore, patients with *CXCL10* rs3921 CG or *CXCL11* rs4619915 AG genotypes had lower odds of significant fibrosis (LSM ≥ 7.1 kPa) [adjusted OR (aOR) = 0.56 (p = 0.008)], advanced fibrosis (LSM ≥ 9.5 kPa) [aOR = 0.55 (p = 0.013)], and cirrhosis (LSM ≥ 12.5 kPa) [aOR = 0.57 (p = 0.051)]. Additionally, we also found that *CXCL9*-*11* polymorphisms were related to lower liver stiffness under a codominant model of inheritance (*data not shown*), being the heterozygous genotypes also protective against hepatic fibrosis.

**Table 4:** Relationship between *CXCL9*-*11* polymorphisms (*CXCL9* rs10336, *CXCL10* rs3921, and *CXCL11* rs4619915) and liver fibrosis in HCV-infected patients

	Unadjusted		Adjusted	
	Exp(B) (95% CI)	p^a^	Exp(B) (95% CI)	p^b^
rs10336 AG				
LSM (kPa)	0.82 (0.73; 0.92)	*0.001*	0.85 (0.76; 0.95)	*0.005*
F ≥ 2 (≥7.1)	0.55 (0.37; 0.83)	*0.004*	0.59 (0.38; 0.91)	*0.016*
F ≥ 3 (≥9.5)	0.48 (0.31; 0.74)	*0.001*	0.54 (0.34; 0.86)	*0.010*
F4 (≥12.5)	0.52 (0.31; 0.89)	*0.016*	0.56 (0.32; 0.98)	*0.043*
rs3921 CG				
LSM (kPa)	0.82 (0.73; 0.91)	*<0.001*	0.84 (0.76; 0.94)	*0.003*
F ≥ 2 (≥7.1)	0.53 (0.35; 0.79)	*0.002*	0.56 (0.36; 0.86)	*0.008*
F ≥ 3 (≥9.5)	0.49 (0.32; 0.76)	*0.002*	0.55 (0.34; 0.88)	*0.013*
F4 (≥12.5)	0.54 (0.31; 0.91)	*0.021*	0.57 (0.33; 1.00)	0.051
rs4619915 AG				
LSM (kPa)	0.82 (0.73; 0.91)	*<0.001*	0.84 (0.76; 0.94)	*0.003*
F ≥ 2 (≥7.1)	0.53 (0.35; 0.79)	*0.002*	0.56 (0.36; 0.86)	*0.008*
F ≥ 3 (≥9.5)	0.49 (0.32; 0.76)	*0.002*	0.55 (0.34; 0.88)	*0.013*
F4 (≥12.5)	0.54 (0.31; 0.91)	*0.021*	0.57 (0.33; 1.00)	0.051

*Exp(B)* geometric mean ratio (GMR) continuous variable or odds ratio (OR) for categorical variables, *95%CI* 95% of confidence interval, *p value* level of significance, *LSM* liver stiffness measure, *kPa* kilopascal, *F* *≥* *2* significant fibrosis, *F* *≥* *3* advance fibrosis, *F4* cirrhosis, *HCV-GT* hepatitis C virus genotype, *CXCL* chemokine (C-X-C motif) ligand

Statistical: ^a^ p values were calculated by univariate regression

^b^p values were calculated by multivariate regression adjusted by the most important clinical and epidemiological characteristics (see “statistical analysis” section). Statistically significant differences are shown in italic

Table 5 shows the relationship between *CXCL9*-*11* polymorphisms and liver stiffness under a recessive inheritance model. The *CXCL9* rs10336 AA, *CXCL10* rs3921 CC and *CXCL11* rs4619915 AA were associated with higher LSM values [aGMR = 1.19 (p = 0.030), aGMR = 1.21 (p = 0.023), and aGMR = 1.21 (p = 0.023), respectively]. Moreover, patients with *CXCL9* rs10336 AA genotype had higher odds of significant fibrosis (LSM ≥ 7.1 kPa) [adjusted OR (aOR) = 1.83 (p = 0.044)] and advanced fibrosis (LSM ≥ 9.5 kPa) [aOR = 1.85 (p = 0.045)]. Furthermore, patients with *CXCL10* rs3921 CC or *CXCL11* rs4619915 AA genotypes had higher odds of advanced fibrosis (LSM ≥ 9.5 kPa) [aOR = 1.89 (p = 0.038)].

**Table 5:** Relationship between *CXCL9*-*11* polymorphisms (*CXCL9* rs10336, *CXCL10* rs3921, and *CXCL11* rs4619915) and liver fibrosis in HCV-infected patients (recessive inheritance model)

	Unadjusted		Adjusted	
	Exp(B) (95% CI)	p^a^	Exp(B) (95% CI)	p^b^
rs10336 AA				
LSM (kPa)	1.21 (1.03; 1.49)	*0.022*	1.19 (1.02; 1.41)	*0.030*
F2 (≥7.1)	2.09 (1.21; 3.63)	*0.009*	1.83 (1.02; 3.32)	*0.044*
F3 (≥9.5)	2.01 (1.15; 3.47)	*0.014*	1.85 (1.02; 3.37)	*0.045*
F4 (≥12.5)	1.81 (0.97; 3.98)	0.062	1.83 (0.94; 3.54)	0.073
rs3921 CC				
LSM (kPa)	1.21 (1.03; 1.44)	*0.019*	1.21 (1.03; 1.42)	*0.023*
F2 (≥7.1)	2.03 (1.16; 3.52)	*0.012*	1.76 (0.97; 3.20)	0.061
F3 (≥9.5)	2.07 (1.18; 3.59)	*0.010*	1.89 (1.04; 3.47)	*0.038*
F4 (≥12.5)	1.86 (0.99; 3.48)	0.053	1.89 (0.97; 3.66)	0.061
rs4619915 AA				
LSM (kPa)	1.22 (1.03; 1.44)	*0.019*	1.21 (1.03; 1.42	*0.023*
F2 (≥7.1)	2.03 (1.16; 3.53)	*0.012*	1.76 (0.97; 3.20)	0.061
F3 (≥9.5)	2.07 (1.19; 3.59)	*0.010*	1.89 (1.04; 3.47)	*0.038*
F4 (≥12.5)	1.86 (0.99; 3.49)	0.053	1.89 (0.97; 3.66)	0.061

*Exp(B)* geometric mean ratio (GMR) for continuous variable or odds ratio (OR) for categorical variables, *95% CI* 95% of confidence interval, *p value* level of significance, *LSM* liver stiffness measure, *kPa* kilopascal, *F* *≥* *2* significant fibrosis, *F* *≥* *3* advance fibrosis, *F4* cirrhosis, HCV-GT hepatitis C virus genotype, *CXCL* chemokine (C-X-C motif) ligand

Statistical: ^a^ p values were calculated by univariate regression

^b^p values were calculated by multivariate regression adjusted by the most important clinical and epidemiological characteristics (see “statistical analysis” section). Statistically significant differences are shown in italic

### *CXCL9*-*11* haplotypes and liver fibrosis

Only three major haplotypes were found (Table 6), which included over 99.3% of patients: GGG, ACA, and AGG. However, we did not found any significant association.

**Table 6:** Association between *CXCL9*-*11* haplotypes and liver fibrosis stage in HCV-infected patients

	*CXCL9*-*11* Haplotypes			Association		p
	rs10336	rs3921	rs4619915	Freq.	aOR (95% CI)	
F ≥ 2 (≥7.1 kPa)	G	G	G	0.581	0.95 (0.70; 1.30)	0.760
	A	C	A	0.401	1.01 (0.74;1.39)	0.929
	A	G	G	0.011	2.50 (0.59;10.5)	0.198
F ≥ 3 (≥9.5 kPa)	G	G	G	0.581	0.97 (0.70;1.36)	0.868
	A	C	A	0.401	1.10 (0.78;1.54)	0.597
	A	G	G	0.011	0.32 (0.04;2.64)	0.226
F4 (≥12.5 kPa)	G	G	G	0.581	0.91 (0.62;1.34)	0.629
	A	C	A	0.401	1.12 (0.76;1.66)	0.557
	A	G	G	0.011	0.58 (0.07;4.85)	0.591

Statistical: p values were calculated by multivariate logistic regression adjusted by the most important clinical and epidemiological characteristics

*95% CI* 95% of confidence interval, *aOR* adjusted odds ratio, *p value* level of significance, *F* *≥* *2* significant fibrosis, *F* *≥* *3* advance fibrosis, *F4* cirrhosis, *HCV-GT* hepatitis C virus genotype, *CXCL* chemokine (C-X-C motif) ligand

## Discussion

In our study, we found that *CXCL9*-*11* polymorphisms, under an overdominant inheritance model, were associated with lower LSM values and fibrosis stage. This association was particularly important with significant fibrosis (F ≥ 2, LSM ≥ 7.5 kPa) and advanced liver fibrosis (F ≥ 3, LSM ≥ 9.5 kPa). To our knowledge, this is the first study that reports a relationship between *CXCL9*-*11* polymorphisms and liver fibrosis in HCV-infected patients. Additionally, this work confirms the previous findings of our group in HIV/HCV-coinfected patients [34]; suggesting that *CXCL9*-*11* polymorphisms may play a key role in the development of liver fibrosis during CHC.

Hepatitis C virus infection is characterized by an increased production of cytokines and chemokines, including the C-X-C motif chemokine receptor 3-associated chemokines [16, 23, 26, 44], which seems to be an important determinant of histological progression in HCV-infected patients [27, 45]. In this context, CXCL9-11 chemokines are expressed on many cell types, and play a crucial role in CHC through the recruitment to the liver parenchyma of inflammatory cells (monocytes/macrophages, activated T lymphocytes, natural killers and dendritic cells). These cells potentially contribute to the host immune response against the HCV, as well as to the hepatic inflammation and the cascade of events that may lead to fibrogenesis and liver disease progression [15, 22, 27, 45].

There is not much available information of the relationship between *CXCL9*-*11* polymorphisms and liver fibrosis in CHC. *CXCL9* variants have been related to liver fibrosis in mice and humans [21]. *CXCL10* rs1439490 (G-201A) polymorphism has been associated with liver disease in patients infected with hepatitis B virus [29]. In a recent article published by our group, we found that *CXCL9* rs10336 AA, *CXCL10* rs3921 CC and *CXCL11* rs4619915 AA genotypes were related to higher likelihood of severity of liver fibrosis in HIV/HCV-coinfected patients under a recessive model [34]. Here, the homozygous genotypes for the minor alleles showed susceptibility to the liver disease. In the current study, we also showed that *CXCL9*-*11* polymorphisms were also related to higher LSM values and higher odds of severity of liver fibrosis under a recessive model of inheritance. However, the overdominant model was the one that fitted best to our data, since it had lower values of AIC and BIC. In the overdominant model, the presence of heterozygous genotypes were linked to protection of severity of the liver disease. Thus, heterozygous individuals have a higher fitness than homozygous subjects (heterozygote advantage), such as in the sickle cell anemia, in which homozygote subjects do not have malaria protection or have dramatic propensity to sickle cell anemia; while heterozygote subjects have fewer physiological effects and partial resistance to malaria [46]. However, we do not have data to explain the mechanism by which this occurs.

The three *CXCL9*-*11* SNPs analyzed in this study are located at the 3′ untranslated region (UTR) of their respective genes. 3′UTR is a putative regulatory region, which could be targeted by microRNAs (miRNAs). These molecules are small non-coding RNAs that regulates expression by binding to its mRNA target site. Thus, polymorphisms in 3′UTR region could create, destroy or modify the efficiency of miRNA binding to the 3′UTR of a gene, resulting in gene dysregulation at posttranscriptional level. In order to investigate whether *CXCL9*-*11* polymorphisms could affect mRNA target sites for miRNAs, we performed an in silico analysis by using PolymiRTS Database 3.0 [47]. We found that rs10336 A and rs4619915 A allele generates two miRNA binding sites for hsa-miR-4519 and hsa-miR-5089-3p respectively, whereas rs10336 G and rs4619915 G allele disrupt these miRNA target sites. The lack of binding of these miRNAs to the region surrounding these variants could lead to higher CXCL9-11 levels and, consequently, could modulate the fibrotic effect of these proteins since serum levels of CXCL9 were positively associated with the severity of liver fibrosis [17]. Additionally, the three *CXCL9*-*11* polymorphisms seem to be implicated in changes in the chromatin state in several immune-related cell lines. Genetic variation in regulatory elements might coordinate changes in chromatic states and gene expression at both local and distal sites [48]. By using rVarBase [49], we detected that rs10336 has been associated with a ‘enhancer’ chromatin state in monocytes, rs3921 to a weak transcription in primary B cells and monocytes, and rs4619915 to weak transcription in monocytes among others. All these findings support the idea that *CXCL9*-*11* polymorphisms could affect the production of the CXCL9-11 chemokine levels. In fact, both *CXCL9* rs10336 TT and *CXCL10* rs3921 *GG* genotypes have also been related to higher cellular expression of CXCL9 and CXCL10 in other infectious disease, such as Chagas infection, where they were also related to a higher severity of chronic Chagas cardiomyopathy [32]. In this regard, our data are in line with data of Nogueira et al. [32], because patients with *CXCL9* rs10336 TT and *CXCL10* rs3921 *GG* genotypes (recessive inheritance model) had higher LSM values and fibrosis stages, possibly due to increased production of these chemokines that could allow the development and maintenance of the infiltration of Th1 cells in liver parenchyma. However, it is necessary to take into account that *CXCL9, CXCL10,* and *CXCL11* genes are clustered at chromosome 4q21.2, within a range of 32 kb [50, 51], where there is a high number of additional SNPs, which may be tightly linked to *CXCL9* rs10336, *CXCL10* rs3921, and *CXCL11* rs4619915 polymorphisms [50, 51]. Thereby, rs10336, rs3921, and rs4619915 polymorphisms could be functionally responsible for the observed effect or could just be polymorphisms in high LD with the causal mutation. Therefore, this association that we observed might be due to the proximity to other SNPs that may modulate *CXCL9*-*11* expression, or a direct effect of *CXCL9*-*11* polymorphisms on *CXCL9*-*11* expression. In this regard, CXCL9-11 levels or CXCL9-11 expression could be very helpful and convincing to reinforce the hypothesis that the observed effect of *CXCL9*-*11* SNPs have a biological effect, but such information were not available in this study.

In this study, we analyzed the association between haplotypes and liver fibrosis stages, but we did not find any significant association. The explanation for this lack of association is possibly due to the association of each individual SNP with liver fibrosis under an overdominant model of inheritance, which assumes that both alleles in heterozygous have the strongest impact over the trait. Furthermore, we also compared allelic and genotypic frequencies of *CXCL9* rs10336, *CXCL10* rs3921 and *CXCL11* rs4619915 polymorphism between HCV-infected patients and healthy people (IBS), and no significant differences were found, indicating that our cohort did not have any significant bias regarding the distribution of *CXCL9*-*11* polymorphisms.

Moreover, in terms of translational research, the discriminating power was low for finding patients with liver fibrosis (data not shown). Complex human diseases are under the control of many genes that contribute each one of them with modest individual effects, and only big effects would be detected in small populations. However, the combination of *CXCL9*-*11* polymorphisms together with other biomarkers (plasmatic, genetic, etc.) could add value to the prediction of the development of advanced fibrosis.

This study has several limitations that must be considered to ensure a correct interpretation of the data. Firstly, this is a cross-sectional study, which entails lack of uniformity. Even with this drawback, the association between *CXCL9*-*11* polymorphisms and liver stiffness detected is strong. Secondly, CXCL9-11 chemokines levels in plasma could not be analyzed because we did not have access to plasma samples. Thirdly, since allele frequencies vary among ethnicities, and our study has been carried out entirely in HCV-infected white Europeans, it would be necessary to perform an independent replication of this study for different ethnic groups. Fourthly, in our study, about 20% of the patients were non-responders to IFN-alpha therapy during the follow-up. However, we did not find any significant effect on liver fibrosis progression in the multivariate analysis. Besides, when we excluded non-responder patients, significant associations between *CXCL9*-*11* were also found (data not shown). Fifthly, time since HCV infection is one of the most important factors determining fibrosis in HCV infected patients, but it is difficult to determine because it involves lead-time bias. In this regard, we did not have access to the date of HCV infection in most patients. Instead, we used the time since HCV diagnosis, which may also accumulate bias due to the variability in time from the HCV infection to the HCV diagnosis. Thus, some patients may have HCV infection a long time before HCV diagnosis, when they show symptoms of chronic liver disease, while some may be diagnosed with HCV infection not long after the onset of infection, especially those who had a regular check-up.

## Conclusion

In conclusion, *CXCL9*-*11* polymorphisms were related to likelihood of having liver fibrosis in HCV-infected patients. Our data suggest that *CXCL9*-*11* polymorphisms may play a significant role against the progression of CHC and could help prioritize antiviral therapy.

## Abbreviations

HCV: hepatitis c virus
CHC: chronic hepatitis c
LSM: liver stiffness measurement
SNPs: single nucleotide polymorphisms
CXCL: CXCL chemokine ligand
HIV: human immunodeficiency virus
IFN: interferon
kPa: kilopascals
F ≥ 2—significant fibrosis: LSM ≥ 7.1 kPa
F ≥ 3—advanced fibrosis: LSM ≥ 9.5 kPa
F4—cirrhosis: LSM ≥ 12.5 kPa
UTR: 3′ untranslated region
IBS: Iberian population in Spain
GLM: generalized linear model
GMR: geometric mean ratio
OR: odds ratio
HWE: Hardy-Weinberg equilibrium
LD: linkage disequilibrium
miRNAs: microRNAs
